# Functional connectivity studies in migraine: what have we learned?

**DOI:** 10.1186/s10194-019-1047-3

**Published:** 2019-11-20

**Authors:** Kirill Skorobogatykh, Willem Sebastiaan van Hoogstraten, Diana Degan, Anastasia Prischepa, Anastasya Savitskaya, Biondo Michela Ileen, Enrico Bentivegna, Iaroslav Skiba, Laura D’Acunto, Livia Ferri, Simona Sacco, Jakob Møller Hansen, Faisal Mohammad Amin, European Headache Federation School of Advanced Studies (EHF-SAS)

**Affiliations:** 1 University Headache Clinic, Moscow, Russia; 2 Department of Neuroscience, Erasmus MC, Rotterdam, The Netherlands; 3 Department of Applied Clinical Sciences and Biotechnology, University of L’Aquila, L’Aquila, Italy; 4 Department of Neurology, Sechenov University, Moscow, Russia; 5 Sapienza University of Rome, Rome, Italy; 6 Internal Medicine Unit, Sant’ Andrea Hospital, Sapienza University of Rome, Rome, Italy; 7 Neurology Department, Military Medical Academy, St. Petersburg, Russia; 8 Clinical Unit of Neurology, Department of Medical Sciences, University Hospital and Health Services of Trieste, University of Trieste, Trieste, Italy; 9 Department of Clinical and Molecular Medicine, Faculty of Medicine and Psychology, Rome, Italy; 10 Clinical Neurology Section, Department of Applied Clinical Sciences and Biotechnology, University of L’Aquila, L’Aquila, Italy; 11 Danish Headache Center, Department of Neurology, Rigshospitalet Glostrup, University of Copenhagen, Valdemar Hansens Vej 5, Glostrup, 2600 Copenhagen, Denmark

**Keywords:** Resting-state fMRI, Functional connectivity, Neuroimaging, Migraine, Headache

## Abstract

**Background:**

Resting-state functional connectivity (FC) MRI has widely been used to understand migraine pathophysiology and to identify an imaging marker of the disorder. Here, we review what we have learned from FC studies.

**Methods:**

We performed a literature search on the PubMed website for original articles reporting data obtained from conventional resting-state FC recording in migraine patients compared with healthy controls or during and outside of migraine attacks in the same patients.

**Results:**

We found 219 articles and included 28 in this review after screening for inclusion and exclusion criteria. Twenty-five studies compared migraine patients with healthy controls, whereas three studies investigated migraine patients during and outside of attacks. In the studies of interictal migraine more alterations of more than 20 FC networks (including amygdala, caudate nucleus, central executive, cerebellum, cuneus, dorsal attention network, default mode, executive control, fronto-parietal, hypothalamus, insula, neostriatum, nucleus accumbens, occipital lobe, periaqueductal grey, prefrontal cortex, salience, somatosensory cortex I, thalamus and visual) were reported. We found a poor level of reproducibility and no migraine specific pattern across these studies.

**Conclusion:**

Based on the findings in the present review, it seems very difficult to extract knowledge of migraine pathophysiology or to identify a biomarker of migraine. There is an unmet need of guidelines for resting-state FC studies in migraine, which promote the use of homogenous terminology, public availability of protocol and the a priori hypothesis in line with for instance randomized clinical trial guidelines.

## Introduction

Pathophysiology of migraine is complex and, so far, no biomarker for any of the phases of this cyclic disease exists. During the last decade, advanced neuroimaging modalities are increasingly used to understand migraine pathophysiology and disease mechanisms in the search for imaging markers of migraine. An often-used imaging technique is the resting-state or the so-called functional connectivity (FC) magnetic resonance imaging (fMRI), which has been applied in increasing number of migraine studies, since the first paper was published in 2011 [1]. Ideally, resting-state FC studies may be used to unveil migraine mechanisms.

The migraine resting-state literature is often analyzed and presented in several different ways, which makes it hard to compare results across studies, and findings are at times difficult to understand and are rarely reproduced. Thus, definitive imaging biomarkers for migraine have still not been identified limiting the usefulness and applicability of FC data.

Still, several well-performed resting-state FC studies and reviews [2] are available but a systematic review of the consistency of findings is missing. In the present review, we wish to provide an overview of all published conventional resting-state FC studies and discuss what we have learned so far based on FC findings.

## Methods

### Literature search

Two authors (JMH and FMA) performed search on the PubMed.com website to identify all original articles with resting-state FC data in migraine patients. The literature search was finalized on Pubmed.com September 20th, 2018. We used the following search terms: #1 resting state fMRI and migraine, #2 functional connectivity and migraine, and #3 functional connectivity fMRI and migraine. The search was restricted to human studies published in English language within 10 years, up to September 20th, 2018. Reviews, pediatric studies, case-reports, all other headache diagnoses and letters were excluded. We also assessed reference lists of the found articles for additional relevant studies. Moreover, we excluded all studies that did not use conventional resting-state analysis but other modalities, e.g. functional connectivity density, Granger causality, amplitude of low-frequency fluctuations, and regional homogeneity. Articles, in which the method was not properly described or if data on the comparison to a non-headache control group was not available were also excluded (expect if migraine attacks were compared to an interictal phase). Finally, studies testing treatment effect were also excluded. These exclusion criteria were chosen to include comparable studies in this review.

### Data extraction

To screen for inclusion and exclusion criteria, the senior authors (JMH and FMA) assessed all abstracts found in the initial search. The selected studies were then sent to the co-authors (KS, WSvH, DD, AP, AS, BMI, EB, IS, LDA, and LF) who then read the text and extracted further information, i.e. origin of study, study population, method and main findings.

### Resting-state functional connectivity MRI

The imaging method is based on blood-oxygen-level dependent (BOLD) recordings of the resting brain (i.e. the person lying in the MRI scanner is relaxing with closed eyes, but not sleeping). Every voxel in the obtained image of the brain emits a signal with a specific frequency. The higher the degree of synchronization of signal frequency between two different voxels, the more functional connected are these voxels, and vice versa. Brain areas displaying a particular level of similarity represent a functional connectivity network. Thus, all areas in the brain are more or less functionally connected to each other. The use of this method depends on the change in the functional connectivity between areas in a network, when measured in two different conditions or population samples.

## Results

**Fig. 1:**
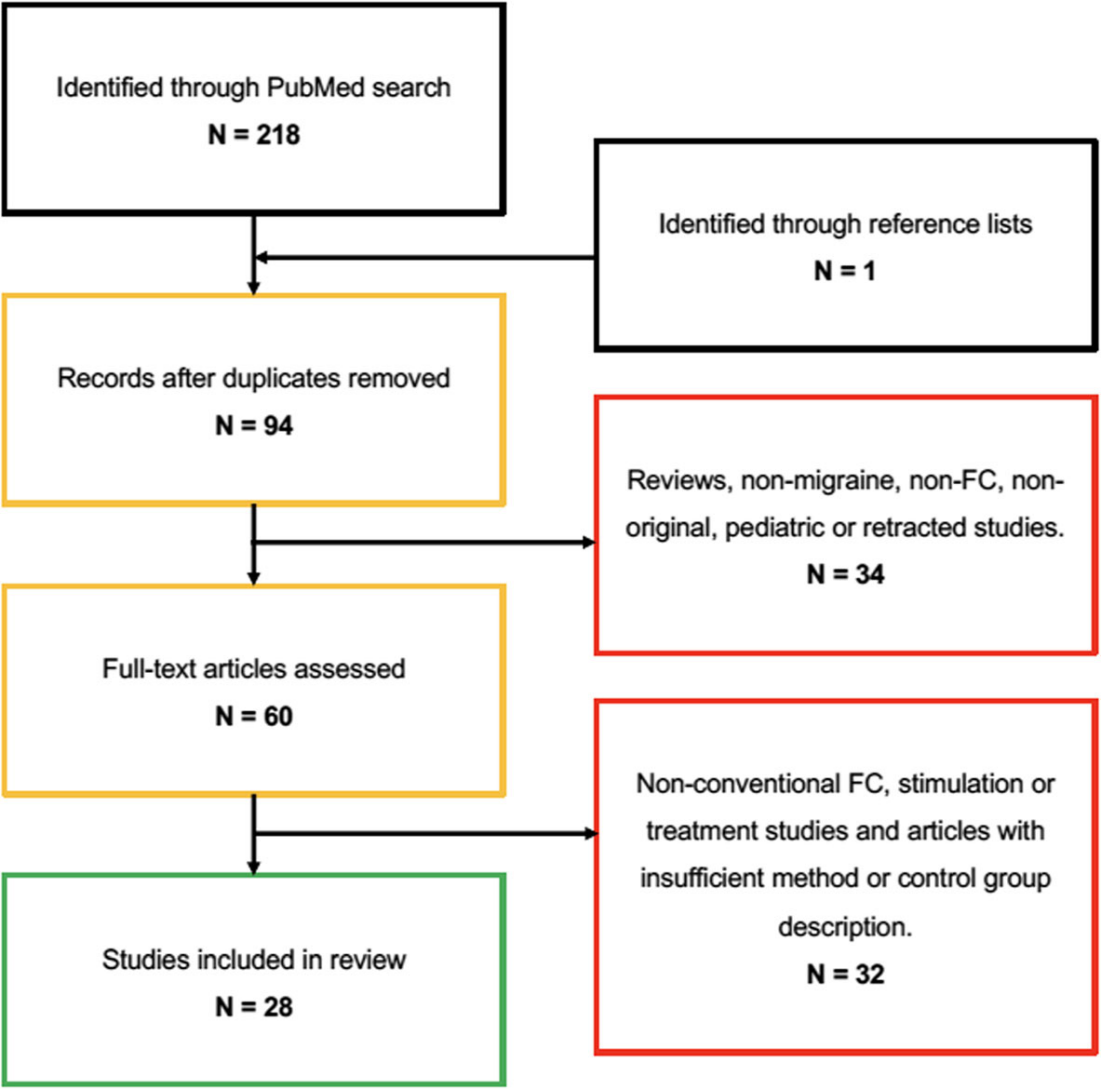
Flow chart of the literature search on functional connectivity (FC) studies in migraine

Our search strategy was finalized September 20th, 2018 and resulted in a total of 219 results, including 94 unique results, from which following were excluded: 15 reviews, 12 stimulation studies, nine non-conventional FC modalities, six examining effect of treatment (acupuncture), five non-migraine studies, five non-FC studies, four non-original articles, one pediatric study, and one study was retracted. Further eight studies were excluded because the method was not properly described or lack of a non-headache control group. One study was subsequently included from the reference lists. We ended up with a total of 28 studies, including 25 during the interictal phase (Table 1) and three during the ictal phase (Table 2) of migraine (Fig. 1). The studies were published between 2011 and 2017 and originated from five different countries, including China = 11; USA = 6; Italy = 6; Denmark = 4; Taiwan = 1.

**Table 1:** Functional connectivity MRI during the interictal phase of migraine compared with non-migraine controls

Study	Population and method	Findings
Mainero, 2011 Ann Neurol [1] Origin: USA.	17 migraine (8 MA and 9 MO) patients were compared to 17 age- and sex-matched controls.	Migraine versus controls PAG: increased FC with right ventrolateral prefrontal cortex, right supramarginal gyrus, right anterior insula, right postcentral gyrus (S1), right thalamus, left angular gyrus, left supramarginal gyrus/parietal operculum (S2), and bilateral precentral gyrus (M1). Decreased FC with right dorsolateral prefrontal cortex, right lateral prefrontal cortex, right anterior cingulate, left dorsomedial prefrontal cortex, left medial prefrontal cortex, and left anterior insula.
	Seed-based approach using FSL. Seeds were used for PAG.	
Yuan K, 2012 PLoS One [3] Origin: China.	21 MO patients were compared to 21 age- and sex-matched controls.	MO versus controls Right ACC: increased FC with bilateral orbitofrontal cortex. Left ACC: increased FC with bilateral orbitofrontal cortex and right dorsolateral prefrontal cortex.
	Seed-based approach using FSL. Seed were bilaterally placed in anterior cingulate cortex (ACC).	
Russo A, 2012 Cephalalgia [4] Origin: Italy.	14 MO patients were compared to 14 age- and sex-matched controls.	MO versus controls FPN: decreased FC with right middle frontal gyrus and right dorsal ACC.
	ICA-based approach using MATLAB to examine fronto-parietal network (FPN).	
Jin C, 2012 NMR Biomed [5] Origin: China.	21 MO were compared with 21 age- and sex-matched controls. Seed-based approach using FSL. Seeds were used for left medial prefrontal cortex (PFC), left dorsal ACC, right occipital lobe, cerebellum and brainstem.	MO versus controls Dorsal ACC: increased FC of bilateral middle temporal lobe, orbitofrontal cortex, and left dorsolateral prefrontal cortex. Right occipital lobe: increased FC of left dorsolateral prefrontal cortex and right middle cingulate cortex. Left medial PFC: increased FC of bilateral dorsolateral prefrontal cortex. Right cerebellum: increased FC with the right medial PFC. Brainstem: no changes were detected.
Xue T, 2012 PLoS One [6] Origin: China.	23 MO patients were compared with 23 age- and sex-matched controls.	MO versus controls Right CEN: increased FC with right middle frontal gyrus and right anterior insula. Left CEN: increased FC with left inferior frontal gyrus. SN: decreased FC with right supplementary motor area. DMN: increased FC with right anterior insula.
	ICA-based and seed-based approach using FSL. Seed were used for default mode network (DMN), central executive network (CEN) and salience network (SN).	
Xue T, 2013 NMR Biomed [7] Origin: China.	18 MO patients were compared with 18 age- and sex-matched controls.	MO versus controls Left ACC: increased FC with bilateral frontal lobe and left parietal lobe. Right thalamus: increased FC with bilateral caudate, left temporal lobe and right putamen. Left PFC: increased FC with right precuneus and bilateral parietal lobe. Right PFC: increased FC with bilateral parietal lobe and left temporal lobe. Right insula: increased FC with left temporal pole, right frontal lobe, and left parietal lobe.
	Seed-based approach using MATLAB. Seeds were used for left ACC, right thalamus, bilateral PFC and right insula.	
Tessitore A, 2013 J Headache Pain [8] Origin: Italy.	20 MO patients were compared with 20 age- and sex-matched controls.	MO versus controls DMN: decreased FC with left superior prefrontal gyrus and left temporal pole.
	ICA-based approach using FSL to identify DMN among a total of 40 networks.	
Schwedt TJ, 2013 Headache [9] Origin: USA.	20 chronic migraine patients were compared with 20 controls.	Chronic migraine versus controls Anterior insula: atypical FC with pulvinar, middle temporal cortex, mediodorsal thalamus, precuneus, PAG, cingulate cortex, and inferior parietal cortex. Amygdala: atypical FC with superior frontal cortex and occipital cortex.
	Seed-based approach using in-house developed software. Seeds were placed in ACC and bilaterally anterior insula and amygdala.	
Hadjikhani, 2013 Cephalalgia [10] Origin: USA.	22 migraine (11 MA and 11 MO) patients were compared to 20 healthy controls.	Migraine versus controls Amygdala: increased FC with anterior insula, secondary somatosensory cortex (S2) and thalamus.
	Seed-based approach using FSL. Seeds were placed in right and left amygdala.	
Yuan K, 2013 J Pain [11] Origin: China.	40 MO patients were compared to 40 age- and sex-matched controls.	MO versus controls Right caudate: increased FC with left insula and left putamen. Left caudate: increased FC with bilateral hippocampal gyrus, left amygdala, left insula and left putamen. Right nucleaus accumbens: increased FC with bilateral parahippocampal gyrus, bilateral ACC, bilateral orbitofrontal cortex, and left posterior cingulate cortex.
	Seed-based approach using FSL. Seeds were used subregions of the basal ganglia (bilateral caudate and right nucleus accumbens).	
Moulton EA, 2014 PLoS One [12] Origin: USA.	12 MO patients were compared with 12 age- and sex-matched controls.	MO versus controls Hypothalamus: increased FC with right precentral gyrus, right middle frontal gyrus, left superior parietal gyrus/supramarginal gyrus, left inferior temporal gyrus, right planum polare, left temporal pole, left middle temporal gyrus, left parahippocampal gyrus, left superior temporal gyrus, bilateral hippocampus, left caudate, right nucleus coeruleus, bilateral pontine nuclei, left cerebellar crus I and II, bilateral cerebellar lobule V, right cerebellar lobules V and VI, left vermal lobules VIIIa and VIIIb and left dentate nucleus. Decreased FC with right precentral gyrus, left frontal pole, left paracingulate gyrus, right superior frontal gyrus, right fusiform gyrus and left lingual gyrus.
	Seed-based approach using FSL. Seeds were used for hypothalamus.	
Tessitore A, 2015 Headache [13] Origin: Italy.	20 MA and 20 MO patients were compared to 20 age- and sex-matched controls.	MA versus controls Right ECN: Decreased FC with right middle frontal gyrus and dorsal ACC.
	ICA-based approach using FSL to identify executive control network (ECN) among a total of 40 networks.	MO versus controls Right ECN: Decreased FC with right middle frontal gyrus and dorsal ACC.
Zhang J, 2016J Headache Pain [14] Origin: China.	22 MO patients were compared with 22 healthy matched controls.	MO versus controls DMN: increased FC with left posterior cingulate cortex and left precuneus.
	ICA-based approach using MATLAB to identify DMN among a total of 20 networks.	
Coppola G, 2016 J Headache Pain [15] Origin: Italy.	18 MO patients were compared to 19 healthy volunteers.	MO versus controls DMN: decreased FC with a network composed of the visuospatial system and medial visual cortical areas.
	ICA-based approach using MATLAB, where a total of 39 networks were identified.	
Niddam DM, 2016 Cephalalgia [16] Origin: Taiwan.	26 MA and 26 MO patients were compared with 26 age- and sex-matched controls.	MA versus controls Left DAN: increased FC with right orbital gyrus, left rectal gyrus, right fusiform gyrus, right middle temporal gyrus and right parahippocampal gyrus. Left SN: decreased FC with bilateral cuneus, left superior occipital gyrus, right lingual gyrus, left fusiform gyrus and left middle temporal gyrus. Right cuneus: increased FC with left cingulate gyrus, bilateral precuneus and bilateral posterior cingulate. Decreased FC with bilateral insula, bilateral middle frontal gyrus, bilateral claustrum, bilateral lentiform nucleus, right inferior frontal gyrus, bilateral cingulate gyrus and bilateral superior frontal gyrus.
	Seed-based approach using MATLAB. Seeds were used for DMN (posterior cingulate cortex), dorsal attention network (DAN) (middle frontal gyrus), SN (anterior insula) and right cuneus.	
		MO versus controls Left DAN: increased FC with right middle temporal gyrus, right parahippocampal gyrus, right middle occipital gyrus and right fusiform gyrus.
		MA versus MO Left SN: decreased FC with bilateral cuneus, bilateral lingual gyrus, left middle temporal gyrus and middle occipital gyrus. Right cuneus: increased FC with left middle frontal gyrus, bilateral cingulate gyrus and right precuneus. Decreased FC with bilateral insula, bilateral claustrum, right lentiform nucleus, right inferior frontal gyrus and left middle frontal gyrus.
Tedeschi G, 2016Cephalalgia [17] Origin: Italy.	20 MA and 20 MO patients were compared to 20 healthy controls.	MA versus MO VN: increased FC with right lingual gyrus.
	ICA-based approach using FSL to examine visual network (VN) among a total of 40 networks.	MA versus MO/controls VN: no changes were found.
Chen Z, 2016 J Headache Pain [18] Origin: China.	18 episodic migraine,16 chronic migraine and 44 medication overuse headache (MOH) + chronic migraine patients were compared to 32 normal controls.	Episodic migraine versus controls Right MdNS: increased FC with right ACC and decreased FC with right insula. Left MdNS: increased FC with right precentral gyrus and decreased FC with right insula.
	Seed-based approach using MATLAB. Seeds were bilaterally placed in the marginal division of neostriatum (MdNS).	Chronic migraine versus controls Right MdNS: increased FC with right middle temporal gyrus. Left MdNS: increased FC with bilateral middle frontal gyrus and left hippocampus.
		MOH + chronic migraine versus controls Right MdNS: increased FC with right interior temporal gyrus and left parahippocampal gyrus. Left MdNS: increased FC with right middle frontal gyrus.
Hodkinson DJ, 2016 eNeuro [19] Origin: USA	40 migraine patients were compared to 40 matched healthy controls.	Migraine versus controls V1: reduced anticorrelation to precuneus and decreased positive correlations to inferior occipital cortex/middle occipital cortex.
	Seed-based approarch using M MATLAB. Seeds were used for networks of vision (V1), audition (primary auditory cortex) and somatosensation (S1).	Primary auditory cortex: reduced anticorrelation to PFC, dorsolateral PFC, precuneus, posterior cingulate cortex and lateral parietal cortex. Decreased positive correlations to insula, opercular cortex, posterior central sulcus and anterior temporal lobe.
		S1: No changes were found.
Androulakis M, 2017 Neurology [20] Origin: USA.	29 chronic migraine patients were compared to 29 age- and sex-matched controls.	Overall connectivity was decreased in all three networks in the chronic migraine group compared to controls.
	Seed-based approach using MATLAB. Seeds were used for SN, CEN and DMN.	Changes were associated with moderate to severe headache and allodynia.
Lo Buono V, 2017 J Headache Pain [21] Origin: Italy.	14 MA patients, 14 MO patients and 14 matched controls.	MA versus MO DMN: increased FC of bilateral central opercular cortex, right insular cortex, bilateral first and second Heschl’s gyrus, left superior temporal gyrus, bilateral lingual gyrus, right occpipital fusiform gyrus, and left occipital pole.
	ICA-based approach using FSL. DMN was examined.	MA versus controls DMN: Increased FC of bilateral Heschl’s gyrus, bilateral planum temporale, and left superior temporal gyrus.
		MO versus controls DMN: Increased FC of bilateral lingual gyrus, occcipital fusiform gyrus, occipital pole, and cingulate gyrus.
Hougaard A, 2017 Eur J Neurol [22] Origin: Denmark.	40 MA patients were compared to 40 age- and sex-matched controls.	Seed-based approach No difference was found in any examined network.
	Seed-based and ICA-based approaches using FSL. Seeds were used for DMN, primary visual cortex, lateral geniculate nucleus, PAG, amygdala, inferior frontal gyrus, superior parietal lobule, inferior parietal lobule, pars opercularis, visual area V2, V3A, V4 and V5.	ICA-based approach No changes were detected in 30 analysed networks.
Chen Z, 2017 J Headache Pain [23] Origin: China.	18 episodic migraine patients (15 MO, 3 MA) were compared with 18 healthy controls.	Episodic migraine versus controls Right ventrolateral PAG: decreased FC with left precentral gyrus. Left ventrolateral PAG: decreased FC with left precentral gyrus, left middle frontal gyrus, left inferior frontal gyrus, bilateral middle temporal gyrus, right superior frontal gyrus and right supplementary motor area. Left dorsolateral PAG: decreased FC with right pars triangularis of inferior frontal gyrus and the medial superior frontal gyrus.
	Seed-based approach using MATLAB. Seeds were used for PAG, incl. Bilateral ventrolateral PAG, lateral PAG, dorsolateral PAG, and dorsomedial PAG.	
Chen Z, 2017 J Headache Pain [24] Origin: China.	18 episodic migraine and 16 chronic migraine patients were compared to 18 normal controls.	Episodic migraine versus controls Left amygdala: increased FC with left middle cingulate gyrus and left precuneus. Right amygdala: no change.
	Seed-based approach using MATLAB. Seed were bilaterally placed in amygdala.	Chronic migraine versus controls Left amygdala: no change. Right amygdala: decreased FC with right inferior occipital lobe and right middle occipital lobe.
		Chronic versus episodic migraine Left amygdala: inferior temporal gyrus, right orbital part of superior frontal gyrus, left fusiform, right postcentral gyrus, left rectus, right amygdala and left precentral gyrus. Right amygdala: inferior temporal gyrus, left middle cingulate gyrus, left orbital part of medial frontal gyrus, left temporal pole, right orbital part of inferior frontal gyrus, right anterior cingulate gyrus and left orbital part of inferior frontal gyrus.
Yu D, 2017 Mol Pain [25] Origin: China.	31 MO patients were compared with 31 age- and education-matched controls.	MO versus controls Right ACC: decreased FC with PFC and posterior cingulate cortex. Left PFC: decreased FC with left insula and posterior parietal cortex.
	Seed-based and ICA-based approaches using FSL. Seeds were used for DMN (medial PFC and posterior cingulate cortex), CEN (dorsloteral PFC and posterior parietal cortex) and SN (frontoinsular cortex and ACC).	No increased FC was found.
Zhang J, 2017 J Neurol [26] Origin: China.	30 MO patients were compared to 31 healthy controls.	MO versus controls Left S1: increased FC with left anterior parietal lobe, right superior parietal lobe, right S1, bilateral premotor cortex, right inferior frontal gyrus, right insula, right temporal lobe, left primary motor cortex and right middle occipital gyrus. Right S1: decreased FC with bilateral premotor cortex, bilateral superior frontal gyrus, bilateral ACC, pons, left insula, bilateral S1, bilateral paracentral lobule, right temporal lobe, right cerebellum lobule VIIIb and left inferior parietal lobule.
	Seed-based approach using MATLAB. Seeds were bilaterally placed in primary somatosensory cortex (S1).	

*MA* Migraine with aura, *MO* Migraine without aura, *FSL* FMRIB Software Library, *FC* Functional connectivity, *ACC* Anterior cingulate cortex, *ICA* Independent component analysis, *CEN* Central executive network, *DAN* Dorsal attention network, *DMN* Default mode network, *ECN* Executive control network, *FPN* Fronto-parietal network, *PAG* Periaqueductal gray, *PFC* Prefrontal cortex, *SN* Salience network, *VN* Visual network, *MdNS* Marginal division of neostriatum

**Table 2:** Functional connectivity MRI during and outside of the ictal phase of migraine

Study	Population and method	Findings
Amin FM, 2016 Neurology [27] Origin: Denmark.	16 MO patients were scanned during and before drug provoked attack. Control group consisted of 15 MO patients who were scanned before and after a vasodilator drug which did not provoke migraine attacks.	During versus before attack SN: increased FC of bilateral opercular part of inferior frontal gyrus. SMN: increased FC of right premotor cortex and decreased of left visual cortex. DMN: increased FC of left primary auditory, secondary somatosensory, premotor, and visual cortices.
	Seed-based approach using MATLAB. Seeds were used for SN, sensorimotor network (SMN) and DMN.	Control group No change was seen between before and after attack recordings.
Hougaard A, 2017 Hum Brain Mapp [28] Origin: Denmark.	16 MA patients were scanned during and outside of a natural provoked attack.	Seed-based approach Attack versus non-attack condition Left pons: increased FC of left primary somatosensory cortex (corresponding to the head and face somatotopic areas). Moreover, increased FC of left superior parietal lobule. Aura-side V5: increased FC with lower middle frontal gyrus (flipped analysis).
	Seed-based and ICA-based approaches using FSL. Seeds were bilaterally placed in cortical visual areas (primary visual cortex, V3, V4, V5), lateral geniculate nucleus, and pons.	ICA-based approach No changes were detected in 56 analysed networks.
Amin FM, 2018 Cephalalgia [29] Origin: Denmark.	17 MO patients were scanned during and outside of a natural provoked attack.	Attack versus non-attack condition Right thalamus: increased FC with left superior parietal lobule, left insular cortex, left primary motor cortex, left supplementary motor area and left orbitofrontal cortex. Moreover, decreased FC with right primary somatosensory cortex and right premotor cortex.
	Seed-based approach using FSL. Seed were bilaterally placed in thalamus, pons, cerebellum crus I, and cerebellum lobule VI.	No change in FC was detected for the remaining seeds.

*MA* Migraine with aura, *MO* Migraine without aura, *FSL* FMRIB Software Library, *FC* Functional connectivity, *ICA* Independent component analysis, *DMN* Default mode network, *SMN* Sensorimotor network, *SN* Salience network

### Interictal migraine versus non-headache controls

Twenty-five published studies reported data comparing interictal migraine with non-migraine non-headache controls. In 12 studies a migraine without aura (MO) population was examined, while pure migraine with aura (MA) was only investigated in a single study. In four studies, data for both MA and MO groups were reported separately, whereas mixed results were reported in the remaining eight studies.

**Fig. 2:**
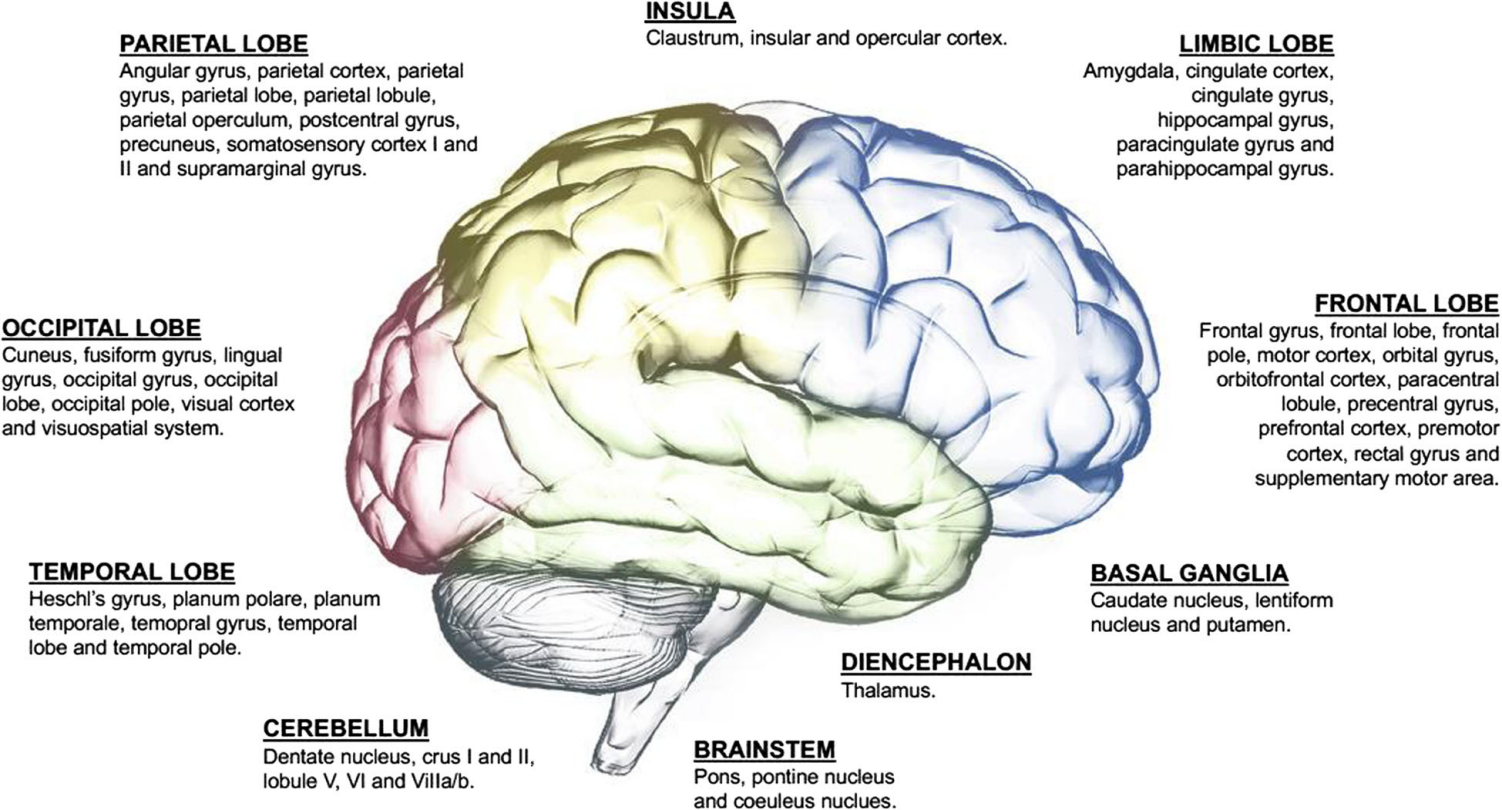
Overview of areas which have affected functional connectivity to 20 different networks reported in a total of 25 studies of interictal migraine patients compared with healthy controls

When comparing migraine patient to controls, the functional connectivity was changed within or with a number of different networks or seed areas: periaqueductal gray network [1, 23], left [3, 7] dorsal [5] and right [3, 25] anterior cingulate cortex, fronto-parietal-network [4], right occipital lobe [5], left medial [5] and bilateral [7] prefrontal cortex, right cerebellum [5], brainstem [5], bilateral central executive network [6, 20], left [16] salience network [6, 20], default mode network [6, 8, 14, 15, 20, 21], right thalamus [7], right [7] and anterior [9] insula, amygdala [9, 10, 24], bilateral caudate [11], right nucleus accumbens [11], hypothalamus [12], right executive control network [13], left dorsal attention network [16], right cuneus [16], visual network [17], marginal division of neostriatum [18], primary visual cortex [19], primary auditory cortex [19] and bilateral primary somatosensory cortex [26]. All areas with abnormal connectivity to the above-mentioned networks are shown in Table 1 and Additional file 1 and Fig. 2.

### Ictal migraine versus non-headache controls

Three conventional resting-state FC studies (one MA and two MO) have been published during compared to outside of migraine attacks. Following networks or areas showed altered connectivity during the attack versus outside of the attack: salience network [27], somatosensory network [27], default mode network [27], left pons [28] and right thalamus [29]. All areas with abnormal connectivity to the above-mentioned networks and areas are shown in Table 2.

## Discussion

Based on this first systematic review of isolated conventional FC studies in migraine, we report that several areas and networks throughout the brain, brainstem and cerebellum showed altered connectivity in interictal and ictal migraine studies.

The findings are very diverse, with change in FC in many area thought to relevant for migraine as well as several other areas. The fact that almost all published studies report changes to some degree in all areas studied makes it difficult to gather the results into a coherent model, of specific activation patterns of activation in migraine.

All included studies (Tables 1 and 2) shared many characteristics; they used a 3 T MRI scanner, same type of patients (either MA or MO according to the International Classification of Headache Disorders criteria) and controls and in addition analyzed data using almost similar approaches (ICA or seed-based) in either the FSL or MATLAB-based software packages. Seed-based analysis can be affected by the chosen seed. Alterations in the default mode network (DMN) is most frequently reported. However, selection of different seed coordinates for DMN could potentially be the reason why FC changes in the DMN are different across studies. The strength of ICA is that it is independent of seed selection and more reproducible findings should be expected. The ICA-approach has been used in 10 studies and even in these studies different findings were reported.

Migraine is a heterogeneous disorder (with different disease duration, attack frequency, co-morbidity, effect of treatment, presence of aura), which might cause variation in results between studies. We did, however, only include studies where headache was diagnosed according to strict and uniform International Classification of Headache Disorders criteria.

In recent resting-state fMRI studies supplementary analyses like the Granger causality [30–32] have been introduced to investigate if FC changes can be linked to migraine phenotypes in the examined populations, but even here findings cannot be reproduced. As it is clear from Additional file 1 the findings are scattered and show very little overlap (Additional file 1). Moreover, none of the reported FC changes may be specific for migraine as other studies reported similar or exact same network changes in several other conditions, including fibromyalgia [33], Parkinsonian syndromes [34, 35] altered consciousness states [36], systemic lupus [37] and chronic hepatitis C virus infection [38]. Thus, it can be suspected that this FC method is at all not reproducible, which may be due to lack of sensitivity and specificity. Furthermore, to the best of our knowledge no sample size or power calculation guidelines are available for resting-state FC, with the consequence that a meaningful sample size for a resting-state FC study remains unknown. To avoid spurious findings, it would be useful to consider either sharing of data or joining patients in multicenter studies to allow for better and more reproducible studies.

As is already the norm for clinical trials, FC studies should be based on publically available protocols. It is also noteworthy that since very few studies report “negative results” or no changes in FC, primary endpoints should be chosen before initiating studies, as is already the case for randomized clinical trials (RCT). The fact that few (if any) results are reproducible, strongly suggest that stricter methodological guidelines for FC studies are warranted.

Almost half of the presented studies included only MO patients which gives a total sum of 348 MO patients, where 120 MA patients can be calculated in our tables. The FC method may be useful for the study of specific sub-types of migraine if these are clearly selected beforehand, preferable based on a calculation of the necessary number of patients, and with a clear hypothesis to be tested.

The FC method is very versatile and may potentially help improve our understanding of underlying disease mechanisms and even define biomarkers or migraine. Based on this systematic review, we suggest that the current lack of uniform study design, a priori hypothesis and diverse analyses and terminology makes it difficult to apply the available data for a coherent understanding of migraine.

## Conclusions

Imaging, including FC studies could potentially help improve our understanding of underlying disease mechanisms, but so far no reproducible biomarkers of migraine have been identified. Future FC studies should either pool existing data to extract information about sub-phenotypes of migraine patients or follow guidelines similar to RCT guidelines in case of design of new FC studies.

## Supplementary information

**Additional file 1: Table S2.** Schematic overview of regions with an altered functional connectivity to the examined networks throughout 25 studies of interictal migraine compared with healthy volunteers

## Data Availability

All included references in the present review article are available on the Internet.

## Abbreviations

ACC: Anterior cingulate cortex
CEN: Central executive network
DAN: Dorsal attention network
DMN: Default mode network
ECN: Executive control network
FC: Functional connectivity
fMRI: Functional magnetic resonance imaging
FPN: Fronto-parietal cortex
FSL: FMRIB Software Library
ICA: Independent component analysis
MA: Migraine with aura
MdNS: Marginal division of neostriatum
MO: Migraine without aura
MOH: Medication overuse headache
PAG: Periaqueductal gray
PFC: Prefrontal cortex
RCT: Randomized clinical trials
S1: Primary somatosensory cortex
SMN: Sensorimotor network
SN: Salience network
VN: Visual network

## References

[1] Mainero C, Boshyan J, Hadjikhani N (2011). Altered functional magnetic resonance imaging resting-state connectivity in periaqueductal gray networks in migraine. Ann Neurol.

[2] Schwedt TJ, Chiang CC, Chong CD, Dodick DW (2015). Functional MRI of migraine. Lancet Neurol.

[3] Yuan K, Qin W, Liu P, Zhao L, Yu D, Zhao L, Dong M, Liu J, Yang X, von Deneen KM, Liang F, Tian J (2012). Reduced fractional anisotropy of corpus callosum modulates inter-hemispheric resting state functional connectivity in migraine patients without aura. PLoS One.

[4] Russo A, Tessitore A, Giordano A, Corbo D, Marcuccio L, De Stefano M, Salemi F, Conforti R, Esposito F, Tedeschi G (2012). Executive resting-state network connectivity in migraine without aura. Cephalalgia.

[5] Jin C, Yuan K, Zhao L, Zhao L, Yu D, von Deneen KM, Zhang M, Qin W, Sun W, Tian J (2013). Structural and functional abnormalities in migraine patients without aura. NMR Biomed.

[6] Xue T, Yuan K, Zhao L, Yu D, Zhao L, Dong T, Cheng P, von Deneen KM, Qin W, Tian J (2012). Intrinsic brain network abnormalities in migraines without aura revealed in resting-state fMRI. PLoS One.

[7] Xue T, Yuan K, Cheng P, Zhao L, Zhao L, Yu D, Dong T, von Deneen KM, Gong Q, Qin W, Tian J (2013). Alterations of regional spontaneous neuronal activity and corresponding brain circuit changes during resting state in migraine without aura. NMR Biomed.

[8] Tessitore A, Russo A, Giordano A, Conte F, Corbo D, De Stefano M, Cirillo S, Cirillo M, Esposito F, Tedeschi G (2013). Disrupted default mode network connectivity in migraine without aura. J Headache Pain.

[9] Schwedt TJ, Schlaggar BL, Mar S, Nolan T, Coalson RS, Nardos B, Benzinger T, Larson-Prior LJ (2013). Atypical resting-state functional connectivity of affective pain regions in chronic migraine. Headache.

[10] Hadjikhani N, Ward N, Boshyan J, Napadow V, Maeda Y, Truini A, Caramia F, Tinelli E, Mainero C (2013). The missing link: enhanced functional connectivity between amygdala and visceroceptive cortex in migraine. Cephalalgia.

[11] Yuan K, Zhao L, Cheng P, Yu D, Zhao L, Dong T, Xing L, Bi Y, Yang X, von Deneen KM, Liang F, Gong Q, Qin W, Tian J (2013). Altered structure and resting-state functional connectivity of the basal ganglia in migraine patients without aura. J Pain.

[12] Moulton EA, Becerra L, Johnson A, Burstein R, Borsook D (2014). Altered hypothalamic functional connectivity with autonomic circuits and the locus coeruleus in migraine. PLoS One.

[13] Tessitore A, Russo A, Conte F, Giordano A, De Stefano M, Lavorgna L, Corbo D, Caiazzo G, Esposito F, Tedeschi G (2015). Abnormal connectivity within executive resting-state network in migraine with aura. Headache.

[14] Zhang J, Su J, Wang M, Zhao Y, Yao Q, Zhang Q, Lu H, Zhang H, Wang S, Li GF, Wu YL, Liu FD, Shi YH, Li J, Liu JR, Du X (2016). Increased default mode network connectivity and increased regional homogeneity in migraineurs without aura. J Headache Pain.

[15] Coppola G, Di Renzo A, Tinelli E, Lepre C, Di Lorenzo C, Di Lorenzo G, Scapeccia M, Parisi V, Serrao M, Colonnese C, Schoenen J, Pierelli F (2016). Thalamo-cortical network activity between migraine attacks: insights from MRI-based microstructural and functional resting-state network correlation analysis. J Headache Pain.

[16] Niddam DM, Lai KL, Fuh JL, Chuang CY, Chen WT, Wang SJ (2016). Reduced functional connectivity between salience and visual networks in migraine with aura. Cephalalgia.

[17] Tedeschi G, Russo A, Conte F, Corbo D, Caiazzo G, Giordano A, Conforti R, Esposito F, Tessitore A (2016). Increased interictal visual network connectivity in patients with migraine with aura. Cephalalgia.

[18] Chen Z, Chen X, Liu M, Liu S, Shu S, Ma L, Yu S (2016). Altered functional connectivity of the marginal division in migraine: a resting-state fMRI study. J Headache Pain.

[19] Hodkinson DJ, Veggeberg R, Kucyi A, van Dijk KR, Wilcox SL, Scrivani SJ, Burstein R, Becerra L, Borsook D (2017) Cortico-cortical connections of primary sensory areas and associated symptoms in migraine. eNeuro 3. 10.1523/ENEURO.0163-16.2016 10.1523/ENEURO.0163-16.2016 PMC5239993 28101529

[20] Androulakis XM, Krebs K, Peterlin BL, Zhang T, Maleki N, Sen S, Rorden C, Herath P (2017). Modulation of intrinsic resting-state fMRI networks in women with chronic migraine. Neurology.

[21] Lo Buono V, Bonanno L, Corallo F, Pisani LR, Lo Presti R, Grugno R, Di Lorenzo G, Bramanti P, Marino S (2017). Functional connectivity and cognitive impairment in migraine with and without aura. J Headache Pain.

[22] Hougaard A, Amin FM, Magon S, Sprenger T, Rostrup E, Ashina M (2015). No abnormalities of intrinsic brain connectivity in the interictal phase of migraine with aura. Eur J Neurol.

[23] Chen Z, Chen X, Liu M, Liu S, Ma L, Yu S (2017). Disrupted functional connectivity of periaqueductal gray subregions in episodic migraine. J Headache Pain.

[24] Chen Z, Chen X, Liu M, Dong Z, Ma L, Yu S (2017). Altered functional connectivity of amygdala underlying the neuromechanism of migraine pathogenesis. J Headache Pain.

[25] Yu D, Yuan K, Luo L, Zhai J, Bi Y, Xue T, Ren X, Zhang M, Ren G, Lu X (2017). Abnormal functional integration across core brain networks in migraine without aura. Mol Pain.

[26] Zhang J, Su J, Wang M, Zhao Y, Zhang QT, Yao Q, Lu H, Zhang H, Li GF, Wu YL, Liu YS, Liu FD, Zhuang MT, Shi YH, Hou TY, Zhao R, Qiao Y, Li J, Liu JR, Du X (2017). The sensorimotor network dysfunction in migraineurs without aura: a resting-state fMRI study. J Neurol.

[27] Amin FM, Hougaard A, Magon S, Asghar MS, Ahmad NN, Rostrup E, Sprenger T, Ashina M (2016). Change in brain network connectivity during PACAP38-induced migraine attacks: a resting-state functional MRI study. Neurology.

[28] Hougaard A, Amin FM, Larsson HB, Rostrup E, Ashina M (2017). Increased intrinsic brain connectivity between pons and somatosensory cortex during attacks of migraine with aura. Hum Brain Mapp.

[29] Amin FM, Hougaard A, Magon S, Sprenger T, Wolfram F, Rostrup E, Ashina M (2018). Altered thalamic connectivity during spontaneous attacks of migraine without aura: a resting-state fMRI study. Cephalalgia.

[30] Wang T, Zhan W, Chen Q, Chen N, Zhang J, Liu Q, He L, Zhang J, Huang H, Gong Q (2016). Altered resting-state ascending/descending pathways associated with the posterior thalamus in migraine without aura. Neuroreport.

[31] Ning Y, Zheng R, Li K, Zhang Y, Lyu D, Jia H, Ren Y, Zou Y (2018). The altered Granger causality connection among pain-related brain networks in migraine. Medicine (Baltimore).

[32] Wang T, Chen N, Zhan W, Liu J, Zhang J, Liu Q, Huang H, He L, Zhang J, Gong Q (2015). Altered effective connectivity of posterior thalamus in migraine with cutaneous allodynia: a resting-state fMRI study with granger causality analysis. J Headache Pain.

[33] Napadow V, Harris RE (2014). What has functional connectivity and chemical neuroimaging in fibromyalgia taught us about the mechanisms and management of ‘centralized’ pain?. Arthritis Res Ther.

[34] Wolters AF, van de Weijer SCF, Leentjens AFG, Duits AA, Jacobs HIL, Kuijf ML (2018). Resting-state fMRI in Parkinson’s disease patients with cognitive impairment: a meta-analysis. Parkinsonism Relat Disord.

[35] Filippi M, Sarasso E, Agosta F (2019). Resting-state functional MRI in Parkinsonian syndromes. Mov Disord Clin Pract.

[36] Heine L, Soddu A, Gömez F, Vanhaudenhuyse A, Tshibanda L, Thonnard M, Charland-Verville V, Kirsch M, Laureys S, Demertzi A (2012). Resting state networks and consciousness: alterations of multiple resting state networks connectivity in physiological, pharmacological, and pathological consciousness states. Front Psychol.

[37] Mikdashi JA (2016). Altered functional neuronal activity in neuropsychiatric lupus: a systematic review of the fMRI investigations. Semin Arthritis Rheum.

[38] Kharabian Masouleh S, Herzig S, Klose L, Roggenhofer E, Tenckhoff H, Kaiser T, Thöne-Otto A, Wiese M, Berg T, Schroeter ML, Margulies DS, Villringer A (2017). Functional connectivity alterations in patients with chronic hepatitis C virus infection: a multimodal MRI study. J Viral Hepat.

